# Antibiotic Management for Early-Onset Sepsis in Neonates With Gestational Ages of ≥ 34 Weeks: The Kaiser Sepsis Calculator Versus the 2010 CDC Guidelines

**DOI:** 10.7759/cureus.63704

**Published:** 2024-07-02

**Authors:** Thu-Tinh Nguyen, Oanh T.H. Nguyen, Mai N Duong, Linh Tran Phuong Giang

**Affiliations:** 1 Pediatrics, University of Medicine and Pharmacy, Ho Chi Minh City, VNM; 2 Neonatology, University Medical Center, Ho Chi Minh City, VNM; 3 Neonatal Intensive Care, Children's Hospital 2, Ho Chi Minh City, VNM; 4 Neonatal Intensive Care, Nguyen Dinh Chieu Hospital, Ben Tre, VNM; 5 Pediatrics, University of Health Sciences, Vietnam National University - Ho Chi Minh City, Ho Chi Minh City, VNM

**Keywords:** sepsis, neonatal early-onset sepsis diagnostic tool, neonatal early-onset sepsis calculator, neonatal early-onset sepsis, neonatal sepsis

## Abstract

Introduction: The traditional approach to neonatal early-onset sepsis (NEOS) management, involving maternal risk factors and nonspecific neonatal symptoms, usually leads to unnecessary antibiotic use. This study addresses these concerns by evaluating the Kaiser sepsis calculator (KSC) in guiding antibiotic therapy for NEOS, especially in high-incidence facilities (over 4/1,000 live births), by comparing it against the 2010 Centers for Disease Control and Prevention (CDC) guidelines for neonates ≥34 weeks with suspected sepsis, thereby emphasizing its implications for personalized patient care.

Methods: This is a prospective observational study. All neonates of 34 gestational weeks or more, presenting with either maternal risk factors or sepsis symptoms within 12 hours of birth, were included in the study. The analysis focused on antibiotic recommendations by the 2010 CDC guidelines versus those by the KSC at presumed (0.5/1,000) and actual (16/1,000) sepsis incidence rates.

Results: NEOS was identified in 14 cases (14.1%). Compared to the KSC, at an incidence rate of 16 per 1,000, the KSC resulted in a significant 32.3% reduction in antibiotic treatment (74 cases (74.7%) vs. 42 cases (42.4%), respectively; p < 0.001). The calculator advised immediate antibiotic utilization for 13 out of 14 (92.9%) diagnosed cases, suggesting further evaluation for the remaining cases. When a presumed incidence of 0.5/1,000 was applied, the KSC indicated antibiotics less frequently than when using the actual rate of 16/1,000 (p<0.001) with two missed NEOS cases.

Conclusions: Using the KSC led to a decrease of 32 cases (32.3%) in unnecessary antibiotic prescriptions compared to adherence to 2010 CDC guidelines. However, setting a presumed incidence below the actual rate risked missing NEOS. The calculator was effective when actual local incidence rates were used, ensuring no missed cases needing antibiotics.

## Introduction

Neonatal early-onset sepsis (NEOS) is a major cause of mortality in neonates, particularly in low- to middle-income countries (LMICs) such as Vietnam [1]. The World Health Organization has emphasized the need for antimicrobial stewardship (AMS) programs to help healthcare providers optimize antibiotic treatment and enhance patient outcomes to prevent an antimicrobial resistance crisis [2]. Early-life antibiotic exposure disturbs the developing microbiome and raises the risk of antimicrobial resistance in addition to increasing rates of diseases, including diabetes, obesity, inflammatory bowel disease, asthma, and allergies [3,4]. Neonatal antibiotic medications are also linked to higher healthcare expenses, decreased breastfeeding rates, longer hospital stays, and mother-newborn separation [5-7].

The diagnostic gold standard, blood culture, has limited sensitivity in LMICs, which hampers the applicability of treatment guidelines from high-income countries (HICs) to LMIC settings [8]. Current guidelines relying on maternal risk factors and neonate symptoms lack the precision for accurate NEOS diagnosis [9-12], leading to the overuse of antibiotics for well-appearing neonates and missed treatment for those who actually require them [13]. Consequently, it is crucial to intensify efforts to reduce unnecessary antibiotic use.

The Kaiser research team introduced an online tool predicting NEOS risk, named Kaiser sepsis calculator (KSC), for neonates born after 34 weeks. The calculator was developed based on a case-control study involving 350 neonates with NEOS and 1,063 control group neonates, with a total of 608,014 live births during the study period [14].

Evaluations and recent reviews highlighted its potential to lower antibiotic use compared to the guidelines of the 2010 Centers for Disease Control and Prevention (CDC) recommendations or local protocols. However, its rate of missed NEOS is still under debate because of the inconsistent data from these studies [15]. Furthermore, most NEOS research originates from HICs, with limited data from LMICs, particularly in regions where incidence exceeds four per 1,000 live births. This scarcity of data has consequently hindered the widespread adoption of the KSC in LMICs. Given the abundance of decision-making tools available, it is crucial to assess not only their application but also their ability to facilitate individualized patient management. This study addresses these concerns by evaluating the KSC in guiding antibiotic therapy for NEOS, especially in high-incidence facilities (over 4/1,000 live births), by comparing it against the 2010 Centers for Disease Control and Prevention (CDC) guidelines for neonates ≥ 34 weeks with suspected sepsis, thereby emphasizing its implications for personalized patient care.

## Materials and methods

Ethical approval

The study was conducted after approval and consent from the Ethics Committee of Nguyen Dinh Chieu Hospital, Ben Tre Province, under decision number 3549/GCN-HĐĐĐ dated October 31, 2021.

Setting

This prospective observational study was conducted for six months, from November 1, 2021, to April 30, 2022, at the level-2 neonatal intensive care unit (NICU) of Nguyen Dinh Chieu Hospital, Vietnam. All neonates of 34 gestational weeks or more, born in the Obstetrics Department of Nguyen Dinh Chieu Hospital and presenting with at least one of the following maternal risk factors (fever ≥ 38°C, chorioamnionitis, positive Group B Streptococcus screening, intrapartum antibiotic prophylaxis < four hours, rupture of membranes ≥ 18 hours) or clinical symptoms related to sepsis within 12 hours of birth, were included in the study.

Chorioamnionitis used in our study was defined as the maternal temperature was ≥ 39.0°C or when the maternal temperature was 38.0-38.9°C and two or more of the following: maternal tachycardia (> 100 beats/min), maternal leukocytosis (white blood cell count > 15,000 cells/mm^3^), uterine fundal tenderness, fetal tachycardia (> 160 beats/min), or purulent or malodorous cervical discharge [16].

Neonates were excluded if (i) mothers had not yet gone into labor due to which we could not apply the predictive variables of KSC, (ii) mothers were using drugs potentially affecting the neonate (addictive substances), (iii) born via cesarean section with endotracheal anesthesia, (iv) presenting with critical congenital heart defects, major structural defects requiring immediate surgical intervention, chromosomal abnormalities, neurological abnormalities, (v) transferred to another facility during the study period, or (vi) informed consent was not obtained.

Exposure

Antibiotic recommendations from the 2010 CDC guidelines versus KSC at actual (16/1,000) and presumed (0.5/1,000) incidence rates.

Outcome

“NEOS” was defined by diagnosis of “sepsis”, which was established with a positive blood culture; “probable sepsis” was identified if the blood culture was negative and there were ≥ three clinical symptoms or ≥ two clinical symptoms in combination with a C-reactive protein (CRP) level > 10 mg/L. “No NEOS” was determined if the blood culture was negative and the neonate was well-appearing [17].

Antibiotic treatment was then determined according to 2010 CDC guidelines and the KSC (https://neocalc.vn/index.php?r=function%2Ffunc13). The KSC recommendations were assessed based on the actual incidence rate of NEOS in our facility in 2021, which was 16 per 1,000 live births, compared to the presumed rate of 0.5 per 1,000 live births recommended by the CDC in the absence of specific facility data. A missed NEOS was identified if the KSC did not have antibiotic recommendations, yet laboratory tests and close monitoring identified neonates as having “sepsis” or “probable sepsis.”

Data collection

Clinical information and laboratory data for both the mothers and the neonates were gathered from medical records. Blood cultures were performed on all neonates with 1 mL of blood drawn from a single site. For each participant, we collected information on both actual and presumed treatment recommendations, the actual treatment being the one currently prescribed (following the 2010 CDC guidelines) and the hypothetical based on the outcomes from the KSC (which was not applied to the participants). To collect information on the hypothetical treatment recommendations of the KSC, we encountered difficulties inputting data into the calculator (https://neonatalsepsiscalculator.kaiserpermanente.org) as the calculator pre-set the incidence rate from 0.1 per 1,000 to 4 per 1,000 live births. In 2021, our unit's actual incidence rate was 16 per 1,000 live births, which was not included in the tool. Consequently, we used Excel to recreate the calculator following the guidance of Puopolo et al., aiming to adjust the ß0 intercept to match our actual incidence rate (since each incidence rate corresponds to a specific ß0 intercept) [14] (see Appendix).

The ß0 intercept here is a concept from the regression model. ß0 intercept refers to a predicted value of the outcome (Y) when all Xi=0. ß0 can be seen as the baseline value (a starting point) of the Y. Otherwise, ßi (regression coefficients) refers to the change in variable Y when the variable Xi changes one unit.

We inputted maternal information and the clinical status of each neonate at two incidence rates of 16 per 1,000 and 0.5 per 1,000 live births and then documented the treatment recommendations from the KSC for each participant.

Statistical analysis

Data analysis was conducted using R software version 4.3.1 (R Development Core Team, Vienna, Austria). Categorical variables were presented as frequency (percentage). Comparisons between two categorical variables were performed using the chi-square test. The level of statistical significance was set at p=0.05 for all tests.

## Results

**Figure 1:**
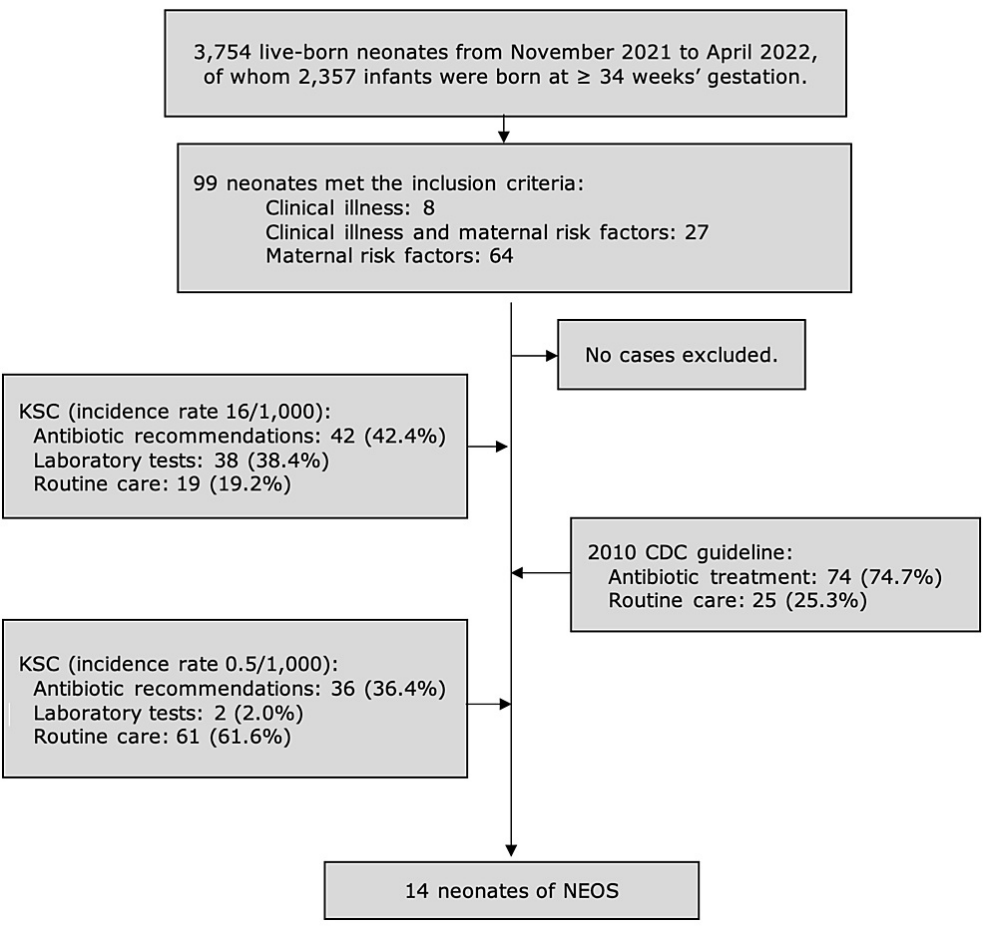
Flowchart displaying the recruitment process and excluded patients in the study. Infants presenting with one of these maternal risk factors (fever ≥ 38°C, chorioamnionitis, positive Group B Streptococcus screening, intrapartum antibiotic prophylaxis < four hours, rupture of membranes ≥ 18 hours) or clinical symptoms related to sepsis within 12 hours of birth. Abbreviations: KSC (Kaiser sepsis calculator); CDC (Centers for Disease Control and Prevention); NEOS (neonatal early onset sepsis)

A total of 99 neonates met the inclusion criteria, with no cases excluded from the study. All 99 neonates received a blood culture. The recruitment process followed the 2010 CDC guidelines and used the Kaiser sepsis calculator at different incidence rates in the study (refer to Figure 1).

Characteristics

The study population of full-term neonates (≥ 37 weeks) accounted for two-thirds of the study population. However, the incidence of probable sepsis was higher among the preterm neonates (34+0/7 weeks to 36+6/7 weeks). The common clinical manifestations included feeding intolerance in 36 cases (36.4%) and respiratory distress necessitating oxygen support in 35 cases (35.4%). There were no cases with positive blood cultures, and diagnoses were primarily based on clinical observation (Table 1).

**Table 1:** Characteristics of the study population (N = 99); data are presented as no. (%) or median (25th percentile; 75th percentile). Abbreviations: NEOS (neonatal early-onset sepsis), GBS (Group B streptococcus), CRP (C-reactive protein)

Characteristics	NEOS (n=14)	No NEOS (n=85)	Total
Maternal risk factors			
Gestational age (weeks)	35.25 (34; 38.7)	39 (37.7; 39.5)	38.7 (36.7; 39.5)
34-< 37	8 (8.1)	18 (18.2)	26 (26.3)
≥37	6 (6.1)	67 (67.6)	73 (73.7)
Highest maternal temperature (°C)	37 (37; 38)	37 (37; 38)	37 (37; 38)
GBS status			
Positive	0	20 (20.2)	20 (20.2)
Negative	0	0	0
Not available	14 (14.1)	65 (65.7)	79 (79.8)
Antibiotics during labor			
Broad-spectrum antibiotics prior to delivery > 4 hours	5 (5.1)	17 (17.2)	22 (22.2)
Broad-spectrum antibiotics prior to delivery 2-3.9 hours	2 (2.0)	4 (4.1)	6 (6.1)
Antibiotics for GBS > 2 hours	0	14 (14.1)	14 (14.1)
No antibiotics, or antibiotics <2 hours	7 (7.1)	50 (50.5)	57 (57.6)
Rupture of membranes before delivery (hours)	6.5 (2.3; 31.9)	2 (0; 7)	2 (0; 8)
≥18 hours	6 (6.1)	7 (7.1)	13 (13.2)
<18 hours	8 (8.1)	78 (78.8)	86 (86.9)
Chorioamnionitis			
Yes	5 (5.1)	34 (34.3)	39 (39.4)
No	9 (9.1)	51 (51.5)	60 (60.6)
Characteristics of neonates			
Clinical symptoms			
Respiratory distress requiring oxygen support	12 (12.1)	23 (23.2)	35 (35.3)
Poor feeding	14 (14.1)	22 (22.2)	36 (36.3)
Altered mental status	11 (11.1)	2 (2.0)	13 (13)
Metabolic acidosis	2 (2.0)	0	2 (2.0)
Diagnosis			
Blood culture negative			
≥ 3 sepsis-related symptoms	10 (10.1)	0	10 (10.1)
≥ 2 symptoms + CRP > 10 mg/L	4 (4.0)	0	4 (4.0)
2 symptoms	0	10 (10.1)	10 (10)
1 symptom	0	26 (26.3)	26 (26)
No symptoms	0	49 (50)	49 (50)
Positive blood culture	0	0	0

Reducing antibiotic use

At an incidence rate of 16 per 1,000 live births in our unit, the KSC recommended 32.3% less antibiotic use than the 2010 CDC guidelines (p < 0.001). However, the utilization of laboratory tests was 38.4% greater in the KSC recommendations than in the 2010 CDC guidelines (p < 0.001) (Table 2).

**Table 2:** Comparison of antibiotic recommendations between the KSC and 2010 CDC guidelines at the incidence rate of 16 per 1,000 live births. N=99, data presented as N (%). *Chi-square test, **: Fisher’s exact test. All tests are statistically significant at a p-value < 0.05. Abbreviations: KSC (Kaiser sepsis calculator), 2010 CDC (Centers for Disease Control and Prevention in 2010)

Recommendations	KSC	2010 CDC	p-value
Antibiotics	42 (42.4)	74 (74.7)	< 0.001*
Laboratory tests	38 (38.4)	0 (0)	< 0.001**
Routine	19 (19.2)	25 (25.3)	0.31*

Minimizing missed NEOS 

Comparison of antibiotic rates between the KSC and 2010 CDC guidelines based on 14 cases of neonatal early-onset sepsis. At an incidence rate of 16 per 1,000 live births, the 2010 CDC guidelines recommended antibiotics for all cases; the KSC recommended antibiotic use for only 13 cases. For the remaining neonates, the KSC estimated a posterior probability of NEOS was 2.1 and recommended additional laboratory tests and close clinical observation every four hours. This case involves a neonate born at 38.3 weeks of gestation, presenting with a maternal fever of 38.5°C, unclear group B streptococcus (GBS) status, and receipt of intrapartum antibiotic prophylaxis 2-3.9 hours before delivery. The neonate exhibited an elevated CRP level of 56.84 mg/L at 12 hours of age, along with symptoms of feeding intolerance and prolonged lethargy exceeding 72 hours. The neonate was subsequently administered antibiotics according to the 2010 CDC guidelines.

When using an incidence rate of 0.5 per 1,000 live births, the KSC missed two NEOS cases. The overlooked neonates included one born at 40 weeks and two days of gestation, with a maternal temperature of 37.5°C, membrane rupture duration of 60 hours, and unclear GBS status. The mother had received broad-spectrum antibiotics for more than four hours before delivery, and the neonate was well-appearing. Using the tool, the calculated disease probability for the neonate was 3.05 (with an incidence rate of 16/1,000), leading to an antibiotic recommendation, and 0.23 (with an incidence rate of 0.5/1,000), prompting the recommendation of routine care. The second neonate, born at 35 weeks and one day of gestation, with the mother having a fever of 38.5°C, membrane rupture for three hours, unclear GBS status, broad-spectrum antibiotics administered to the mother for less than two hours before delivery, and the neonate being well-appearing, had a disease probability at an incidence rate of 16/1,000 live births calculated to be 6.28, leading to an antibiotic recommendation, and 0.19 at an incidence rate of 0.5/1,000 live births, resulting in recommendation for routine care (Table 3).

**Table 3:** Comparison of antibiotic rates using the KSC at incidence rates of 16/1,000 and 0.5/1,000 live births. N=99, data presented as no. (%). *Chi-square test. Statistically significant at a p-value < 0.05. Abbreviations: KSC (Kaiser sepsis calculator), NEOS (neonatal early-onset sepsis)

Recommendations by KSC	NEOS (n=14)	No NEOS (n=85)	Total	p-value*
Incidence rate 16/1,000				
Antibiotics	13 (13.1)	29 (29.3)	42 (42.4)	< 0.001
Laboratory tests	1 (1.0)	37 (37.4)	38 (38.4)	
Routine care	0 (0)	19 (19.2)	19 (19.2	
Incidence rate 0.5/1,000				
Antibiotics	11 (11.1)	25 (25.3)	36 (36.4)	< 0.001
Laboratory tests	1 (1.0)	1 (1.0)	2 (2.0)	
Routine care	2 (2.0)	59 (59.6)	61 (61.6)	

## Discussion

Reducing antibiotic use

Our study, a significant contribution to the field, revealed a 32.3% reduction in antibiotic prescription rates for neonates when using the KSC, a substantial improvement compared to the 2010 CDC guidelines. This finding is in line with a systematic review that also recognized the KSC's effectiveness in reducing antibiotic usage, surpassing other current guidelines such as CDC/American Academy of Pediatrics (AAP) [15] or National Institute for Health and Care Excellence (NICE) guidelines [18]. The variations in outcomes across studies can be attributed to differences in incidence rates, sample sizes, selection criteria, comparison protocols, or research designs. However, the majority of studies consistently demonstrated statistically significant lower initial antibiotic prescribing rates when employing the KSC, highlighting its potential to reduce unnecessary antibiotic use for neonates [15].

The missed NEOS

Our study assessed the missed NEOS of KSC for 14 neonates with NEOS. While the 2010 CDC guidelines recommended antibiotics for all cases, the KSC recommended antibiotic use for only 13 cases. For the remaining neonates, the KSC estimated a posterior probability of NEOS was 2.1 and recommended additional laboratory tests and close clinical observation every four hours (Table 2). This case was subsequently administered antibiotics according to the 2010 CDC guidelines. This case underscores the differing antibiotic prescribing approaches of the two guidelines; the KSC promotes a more conservative treatment strategy, delaying antibiotics unless significant maternal risk factors are present and emphasizing the need for additional laboratory tests and thorough clinical monitoring [9,19].

The second difference involves maternal chorioamnionitis. The 2010 CDC guidelines recommended antibiotics for chorioamnionitis, confirmed histologically or clinically, without considering the neonate's condition [9]. In contrast, the KSC uses the mother's highest temperature intrapartum as one of five risk factors [14]. The diagnosis of clinical chorioamnionitis can be challenging and subjective, often relying on the obstetrician's judgment [9]. The prescription of antibiotics, based on the recognition of maternal chorioamnionitis as a significant risk factor for NEOS, led clinicians to adopt a low threshold for administering antibiotics to neonates. Consequently, the antibiotic recommendation rate of the 2010 CDC guidelines is quite high if based solely on this diagnosis. In our study of 39 neonates whose mothers had clinical chorioamnionitis and were recommended for antibiotic treatment, 34/39 (87.2%) were well-appearing and excluded from sepsis, discontinued antibiotics after 48 hours, and were observed for another 48 hours before discharge (Table 1). However, the KSC would have recommended antibiotics for only 14 of these 39 neonates (35.9%). Our findings align with the study by Money et al. [20], which demonstrated the application of the calculator in well-appearing neonates born to mothers with chorioamnionitis, significantly reducing the antibiotic usage rate from 99% to 2.5%. The question arises whether immediate antibiotic prescription or delayed treatment, allowing for further testing and clinical monitoring with antibiotics prescribed only as clinical signs clarify, is the more rational choice for these clinically equivocal neonates. Compared to the 2010 CDC guidelines, which had antibiotic recommendations for 74 neonates to manage 14 NEOS cases, the KSC advises antibiotics for 42 neonates.

The rate of missed NEOS depends on the incidence rate of NEOS used by the calculator to estimate disease probability. The KSC is calibrated for incidence rates between 0.1 and 4 per 1,000 live births, limiting its use in facilities with higher rates. Lower incidence rates typically result in fewer antibiotic recommendations due to lower calculated probabilities of NEOS. There's a risk of overlooking neonates needing antibiotics when using incidence rates lower than the actual incidence rate. Thus, using accurate incidence rates for each facility is crucial. We compared the antibiotic recommendation rates of the calculator at two incidence rates: 0.5/1,000 (as suggested by the CDC when unit-specific incidence rates are unavailable) and 16/1,000 live births (the actual rate observed at our facility). Results showed a lower antibiotic recommendation rate at the 0.5/1,000 incidence rate; however, at this rate, the calculator failed to identify two neonates at risk for NEOS.

This outcome aligns with Laccetta et al.'s study, comparing the calculator at incidence rates of 0.1/1,000 and 2/1,000 live births, noting fewer antibiotic recommendations at the lower rate but missing one neonate [21]. According to Sloane et al., applying the calculator to neonates born to mothers with chorioamnionitis showed even more significant differences, as these are high-risk participants for sepsis. The authors noted a lower antibiotic recommendation with 289 neonates (at an incidence rate of 0.5/1,000) compared to 533 neonates (at an incidence rate of 4/1,000), but the calculator missed two neonates with positive blood cultures at 0.5/1,000 incidence rate [22]. In a recent prospective study by Jessica et al. [23], comparing the KSC with the National Institute for Health and Care Excellence (NICE) guidelines CG149, seven out of 8,856 live births had positive blood cultures, indicating an incidence rate of 0.8 per 1,000. Initially, with an incidence rate of 0.5 per 1,000, the calculator suggested antibiotics for two neonates, lab tests for two, clinical observation for two, and routine care for one. Upon adjusting to an incidence rate of 0.8 per 1,000, recommendations intensified: two initially recommended for testing were moved to antibiotic treatment, and one under observation was switched to testing. At the same time, the other remained under routine care. Of these seven neonates with positive blood cultures, only one case was in the “clinical illness” category; two cases were in the “equivocal”, and three cases were considered “well-appearing”. This research underscores the calculator's limitations in guiding treatment for neonates with minimal or no symptoms, as it relies on maternal risk factors and unit-specific incidence rates. Consequently, concerns persist regarding missed cases with lower-than-actual incidence rates or overprescribing antibiotics with higher-than-actual rates, reducing the calculator's effectiveness. However, the calculator’s safety is enhanced when aligned with the unit's actual incidence rates.

Limitations of the study

The absence of positive blood culture results, the definitive gold standard for neonatal sepsis diagnosis, was noted in our study. Nevertheless, we employed clinical diagnostic criteria based on Modi et al. [17] with equivalent specificity to positive blood cultures and superior sensitivity, rendering this criterion an acceptable alternative.

## Conclusions

Compared to the 2010 CDC guidelines currently implemented in our unit, the KSC has demonstrated the reducing unnecessary antibiotic usage for neonates. The study also indicates the calculator’s prudent approach towards neonates with ambiguous clinical presentations, advocating for further laboratory tests and close monitoring at four-hour intervals. Adjustments for actual incidence rates were imperative to prevent missed antibiotic requirements. Nonetheless, this calculator serves as a preliminary measure, with thorough observation of clinical progression being essential for promptly identifying clinical deterioration and carefully prescribing antibiotics.

## Appendices

Adjusting the Kaiser sepsis calculator (KSC)

The Rationale for Adjusting the KSC

The Kaiser sepsis calculator (KSC) restricts input to incidence rates between 0.1 and 4 per 1,000 live births. This rate does not correspond to our unit's incidence rate of neonatal early-onset sepsis (NEOS) ≥ 34 weeks of gestational age, measured at 16 per 1,000 live births. Different incidence rates will result in varying "cut-off points" in the logistic regression model of predictive factors. For two subjects with the same risk factor, differing incidence rates will lead to differing risks of NEOS and, consequently, differing treatment recommendations.

Description of the KSC

The calculator was developed based on a case-control study involving 350 infants with NEOS (diagnosed through positive blood cultures) and 1,063 control group infants, with a total of 608,014 live births during the study period. Maternal risk factors were documented, analyzed for their association with the likelihood of NEOS, and incorporated into the following logistic regression model (Table 5):

Risk = 47.8398 + [0.8680\times(tempimp)] - [6.9325\times(ga4mdlng)] + [0.0877\times(ga4mdlng_sq)] + [1.2256\times(romimp)] - [1.0488\times(approptx1)] - [1.1861\times(approptx2)] + [0.5771\times j_gbscar(+)] + [0.0427\times j_gbscar(u)].

Where: 

47.8398: intercept, 

tempimp: highest maternal temperature, 

ga4mdlng: gestational age, 

ga4mdlng_sq: (gestational age)^2^, 

romimp: rupture of membranes before delivery, 

approptx1: specific anti-Group B Streptococcus (GBS) antibiotics ≥2 hours before delivery OR any antibiotics from 2 to 3.9 hours before delivery, 

approptx2: broad-spectrum antibiotics  4 hours before delivery, 

j_gbscar (+): positive GBS in mother, 

j_gbscar(u): GBS status in mother not available.

And then, calculations were performed step-by-steps.

**Table 4:** Definitions of variables in the KSC. Abbreviations: GBS (Group B Streptococcus), LR (Likelihood ratio)

Variables	Definition	Coefficient
Maternal temperature (°C)	the mother's highest recorded temperature; if not available, use the temperature at admission	+0.868
Rupture of membranes (hours)	[Rupture of membranes before delivery + 0,05]^0.2^	+1.2256
Gestational age	Calculate in weeks + days/7	-6.9325
(Gestational age)^2^	Calculate in weeks + days/7	+0.0877
Antibiotics during delivery	Antibiotics against GBS are administered ≥ 2 hours before delivery or any antibiotics administered 2 - 3.9 hours before delivery	-1.0488
	Broad-spectrum antibiotics 4 hours before delivery	-1.1861
GBS	Positive	+0.5771
	Not available	+0.0427
Clinical category		
“Clinical Illness”: one of the symptoms	NCAP/HFNC/mechanical ventilation is required (outside the delivery room).	LR=21.2
	Unstable hemodynamics (requires vasopressors)	
	Neonatal neurological disease or deterioration of awareness: (i) Seizures, (ii) Apgar score < 5 at 5 minutes, (iii) Oxygen supplementation needed for ≥ 2 hours to maintain SaO2 > 90% (outside the delivery room).	
“Equivocal”	Prolonged physiological abnormalities ≥ 4 hours: (i) Heart rate ≥ 160 beats per minute; (ii) Respiratory rate ≥ 60 breaths per minute; (iii) Unstable temperature ≥ 38.0°C or < 36.0°C; (iv) Respiratory distress (grunting, chest retractions, nasal flaring) without the need for oxygen supplementation	LR=5
	≥ 2 prolonged physiological abnormalities lasting ≥ 2 hours (abnormalities may not be continuous): (i) Heart rate ≥ 160 beats per minute; (ii) Respiratory rate ≥ 60 breaths per minute; (iii) Unstable temperature ≥ 38.0°C or < 36.0°C; (iv) Respiratory distress (grunting, chest retractions, nasal flaring) without the need for oxygen supplementation	
“Well Appearing”	No prolonged physiological abnormalities	LR=0.41

Steps of the KSC [14]

Step 1: Calculate NEOS risk per/1,000 births (Prior Probability of NEOS):

= β_{0} + (0.8680 \times \text{maternal T}_{0}) - (6.9325 \times \text{gestational age}) + (0.0877 \times \text{gestational age}^{2}) + (1.2256 \times text{rupture of membranes}) - (1.0488 \times \text{antibiotics against GBS ≥ 2 hours before delivery OR any antibiotics administered 2 - 3.9 hours before delivery}) - (1.1861 \times \text{broad-spectrum ≥ 4 hours before delivery}) + (0.5771 \times \text{positive GBS}) + (0.0427 \times \text{unclear GBS status}).

Step 2: Calculate Prior Odds

Prior Odds = \frac{Prior Probability of NEOS}{1 - Prior Probability of NEOS}

Step 3: Calculate Posterior Odds

Posterior odds = Prior Odds \times Likelihood ratio

·       Likelihood ratio “Clinical Illness”  = 21.2 

·       Likelihood ratio “Equivocal”        = 5 

·       Likelihood ratio “Well Appearing” = 0.41

Step 4: Posterior Probability of NEOS (NEOS Risk after Clinical Exam)

Posterior Probability=\frac{Posterior Odds}{1+ Posterior Odds}

Step 5: Final recommendations

·       NEOS Risk after Clinical Exam < 1: Routine care

·       NEOS Risk after Clinical Exam 1 - < 3: Testing and monitoring every 4 hours within 24 hours.

·       NEOS Risk after Clinical Exam 3: antibiotics

Adjusting the Cut-off Point According to the Current Incidence Rate

The cutoff point "47.8398" in the calculator is based on the population in the study of over 350 sick children/1,043 controls. However, the actual incidence rate in the population would be the number of sick children/the number of live births during the same period. With 608,014 live births during the study period, the incidence rate would be 350/608,014 = 0.6/1,000 live births. The author adjusts the cutoff point to correspond to an equivalent current incidence rate of 40.7489. In a different population group, when introduced into the regression model, the cutoff point will change. Therefore, the author recommends adjusting the cutoff point to match the current incidence rate of our unit when applying the calculator [14].

Some of the cutoff points the calculator uses for different current incidence rates include (i) an incidence rate of 0.3/1,000 live births - 40.0528, (ii) an incidence rate of 0.4/1,000 live births - 40.3415, (iii) an incidence rate of 0.5/1,000 live births - 40.5656, and (iv) a incidence rate of 0.6/1,000 live births - 40.7489.

The adjustment of the threshold points is carried out according to the following formula:

β_{0} = ln [(\frac{p}{1-p}) \times (\frac{1-0.0006}{0.0006})] + 40.7489

Where p is the current incidence rate of each unit.

Formulating the adjustment formula for threshold points based on the current incidence rate

The adjustment formula for threshold points based on the current incidence rate is derived as follows:

Starting from the logistic regression formula: 

ln\frac{p}{1-p} = β_{0} + Σβ(X) (1) (β0 depends on p)

The values of p1 and p2 are substituted into (1):

                  ln\frac{p_{1}}{1-p_{1}} = β_{0_{1}} + Σβ(X) (2)

                  ln\frac{p_{2}}{1-p_{2}} = β_{0_{2}} + Σβ(X) (3)

Subtracting (3) from (2):

ln (\frac{p_{1}}{1-p_{1}}) - ln(\frac{p_{2}}{1-p_{2}}) = (β_{0_{1}} +Σβ(X) - (β_{0_{1}} +Σβ(X) = β_{0_{1}} - β_{0_{2}}

=>ln [(\frac{p_{1}}{1-p_{1}}) \times (\frac{1-p_{2}}{p_{2}})] = β_{0_{1}} - β_{0_{2}} (4)

With = 0.6/1,000 = 0.0006, we have the corresponding threshold point = 40.7489.

Substituting into formula (4), we have:

ln [(\frac{p_{1}}{1-p_{1}}) \times (\frac{1-0.0006}{0.0006})] = β_{0_{1}} - 40.7489

β_{0_{1}} = ln [(\frac{p_{1}}{1-p_{1}}) \times (\frac{1-0.0006}{0.0006})] + 40.7489

We calculated the appropriate threshold point for an incidence rate of 16/1,000 live births to be 44.0478.
